# Sixty-One Volatiles Have Phylogenetic Signals Across Bacterial Domain and Fungal Kingdom

**DOI:** 10.3389/fmicb.2020.557253

**Published:** 2020-09-30

**Authors:** Moamen M. Elmassry, Mohamed A. Farag, Robert Preissner, Björn-Oliver Gohlke, Birgit Piechulla, Marie C. Lemfack

**Affiliations:** ^1^Department of Biological Sciences, Texas Tech University, Lubbock, TX, United States; ^2^Department of Pharmacognosy, Faculty of Pharmacy, Cairo University, Giza, Egypt; ^3^Department of Chemistry, School of Sciences and Engineering, The American University in Cairo, New Cairo, Egypt; ^4^Institute of Physiology and Science-IT, Charité – Universitätsmedizin Berlin, Berlin, Germany; ^5^Institute of Biological Science, University of Rostock, Rostock, Germany

**Keywords:** microbial volatile organic compounds, mVOC, bacteria, fungi, phylogenetic signal, markers, multivariate microbial volatilomes phylogenetic analysis, MVOCs

## Abstract

Microorganisms are diverse in their genome sequences and subsequently in their encoded metabolic pathways, which enabled them to adapt to numerous environmental conditions. They produce thousands of small molecules, many of which are volatiles in nature and play important roles in signaling in intra- and inter-species to kingdom and domain interactions, survival, or virulence. Many of these compounds have been studied, characterized, and organized in the mVOC 2.0 database. However, such dataset has not been investigated comprehensively in terms of its phylogeny to determine key volatile markers for certain taxa. It was hypothesized that some of the volatiles described in the mVOC 2.0 database could function as a phylogenetic signal since their production is conserved among certain taxa within the microbial evolutionary tree. Our meta-analysis revealed that some volatiles were produced by a large number of bacteria but not in fungal genera such as dimethyl disulfide, acetic acid, 2-nonanone, dimethyl trisulfide, 2-undecanone, isovaleric acid, 2-tridecanone, propanoic acid, and indole (common bacterial compounds). In contrast, 1-octen-3-ol, 3-octanone, and 2-pentylfuran (common fungal compounds) were produced primarily by fungal genera. Such chemical information was further confirmed by investigating genomic data of publicly available databases revealing that bacteria or fungi harbor gene families involved in these volatiles’ biosynthesis. Our phylogenetic signal testing identified 61 volatiles with a significant phylogenetic signal as demonstrated by phylogenetic *D* statistic *P*-value < 0.05. Thirty-three volatiles were phylogenetically conserved in the bacterial domain (e.g., cyclocitral) compared to 17 volatiles phylogenetically conserved in the fungal kingdom (e.g., aristolochene), whereas 11 volatiles were phylogenetically conserved in genera from both bacteria and fungi (e.g., geosmin). These volatiles belong to different chemical classes such as heterocyclic compounds, long-chain fatty acids, sesquiterpenoids, and aromatics. The performed approaches serve as a starting point to investigate less explored volatiles with potential roles in signaling, antimicrobial therapy, or diagnostics.

## Introduction

Microorganisms are among the most successful organisms in surviving on earth at different environmental conditions and under different stresses (Hengge and Storz, 2011). One of their most impressive attributes for such adaptation tactics lies in their extensive metabolic diversity, which enabled them to synthesize a repertoire of organic compounds (Fondi et al., 2009; Wilmes et al., 2009). These organic compounds serve numerous functions to the individual producers and/or the whole community, e.g., as signaling molecules at the intra- and inter-species to kingdom and domain levels (Ryu et al., 2003; Kai et al., 2009). A significant number of such organic compounds have low molecular mass (100–300 Daltons), typically small compounds (up to C_20_), often with low boiling point, and high vapor pressure, classically denoted as “volatile organic compounds” (VOCs) (Schulz and Dickschat, 2007).

The analysis of microbial VOCs (mVOCs) is complicated by virtue of their chemical complexity and the growth medium components used for bacterial growth (Schulz and Dickschat, 2007). The VOC profiles from clinical and food samples are generally more complex than those derived from pure bacterial cultures grown *in vitro*, which can be considered artificial (Farag et al., 2017). Improved analytical methods are thus needed to ensure comprehensive detection and determination of bacterial volatile profiles under different conditions. Most commonly employed methods for airborne volatile analysis from microbes rely on headspace analysis, in which VOCs from a dynamic airflow over a culture are bound onto an adsorbent filter and then released by rinsing the filter with organic solvents (Timm et al., 2018). Additionally, volatiles can also be sampled in the absence of airflow, using a solid-phase microextraction (SPME) fiber, followed by direct analysis in a heated gas chromatography (GC) (Farag et al., 2013). For a detailed review on mVOC detection methods, please refer to the review by Audrain et al. (2015).

Over 2,000 mVOCs have been detected from around 1,000 bacterial and fungal species, which have been systematically organized in the mVOC 2.0 database^1^ (Lemfack et al., 2014, 2018). In this database the user can search using numerous features or options such as mass spectrum, Kovat’s index, compound class, chemical ID, etc. However, no comprehensive analysis has been performed to investigate the presence or absence of any VOC pattern in such volatilome profile datasets or search for fingerprint VOCs among the different bacterial and fungal taxa. Consequently, the major goal of this study was to test for potential phylogenetic signals in the bacterial and fungal volatilomes. The approach employed is the phylogenetic signal testing, which is defined as the affinity of related taxa to resemble each other in a trait (i.e., production of a specific volatile or set of volatiles) more than they resemble other taxa randomly drawn from the phylogenetic tree (Münkemüller et al., 2012). Such an approach has been thoroughly utilized and successfully used in ecological context to identify phylogenetic patterns in many binary and continuous traits. This was exemplified by numerous groups in the identification of volatile emissions from various eukaryotic species (e.g., identifying volatile terpenes in tropical tree species) (Fritz and Purvis, 2010; Suinyuy et al., 2013; Courtois et al., 2016; Choudoir et al., 2019; Friberg et al., 2019; Hunt et al., 2019).

## Materials and Methods

### Dataset Preparation

A published dataset of volatiles was used in this study. This dataset was obtained from the mVOC 2.0 database^2^ (Lemfack et al., 2014, 2018), which included volatiles collected from ca. 300 original publications. In the original publications, volatile detection methodology was non-standardized and differed significantly in the sample preparation, collection, and analysis. Moreover, the volatilome size was skewed toward certain species or even strains rather than others (e.g., due to their medical or industrial importance). Because of these limitations and to reduce the number of false-positive results, we agglomerated the dataset at the genus level and analyzed the dataset in a qualitative matter (i.e., volatiles present or absent). Furthermore, compounds’ stereochemistry was not considered because different studies were not consistent in detecting the different isomers of compounds. Genera and volatile names were carefully and manually checked for typos, then duplicates were removed. Finally, several misannotated volatiles were excluded from the analysis. This significantly reduced the size of the dataset to 25% of its original volatiles number finally including a total of 474 volatiles across 221 genera. All following analyses were performed in the R Statistical Environment (R Core Development Team^3^) version 3.6.2.

### Phylogenetic Reconstruction and Visualization

Phylogenetic tree of microorganisms, based on the quality checked and aligned ribosomal (r)RNA sequences, was obtained from the SILVA rRNA database project (SILVA SSU release 132^4^) (Quast et al., 2013; Yilmaz et al., 2014). The phylogenetic tree was reconstructed using R packages *adephylo* (Jombart et al., 2010), *phylobase*^5^, *phylosignal* (Keck et al., 2016), *ape* (Paradis et al., 2004), and *phytools* (Puckridge et al., 2013). The phylogenetic tree was pruned to taxa of which mVOCs were included in this analysis. Finally, the phylogenetic tree was visualized and annotated using the Interactive Tree of Life (iTOL) version 5.6.1 (Letunic and Bork, 2019).

### Multivariate Analysis

Multidimensional scaling (MDS) plot was generated using R packages *vegan*^6^ *magrittr*^7^, *dplyr*^8^, and *ggpubr*^9^. Distance matrix was computed using binary method due to the rather qualitative nature of the data, with each volatile designated by a 0 in case of being absent versus 1 when present. The R package *vegan* was used to test for statistical differences in the volatilomes between taxa by implementing permutational analysis of variance (ANOVA) following 999 permutations.

### Measurement of Phylogenetic Signal of Individual Volatiles

To test for a phylogenetic signal for binary traits, we used phylogenetic D statistic for each volatile found in more than one species (Fritz and Purvis, 2010). This index was calculated using the R package *caper*^10^. The D statistic is equal to 1 if the observed binary trait has a phylogenetically random distribution across the tips of the phylogeny and equal to 0 if the observed trait is phylogenetically clustered as if it had been evolved by Brownian motion (null expectation). Using 1,000 permutations, a *P*-value is generated to test whether the observed value of *D* is significantly different from 1 (a random distribution) or 0 (the null expectation). Volatiles with a *P*-value < 0.05 were deemed significant.

### Volatiles Chemical Classification

To consistently annotate volatiles with their appropriate chemical class, first, we used PubChem to retrieve the corresponding IUPAC name^11^ (Kim et al., 2019). Then, web-based application, ClassyFire^12^ was employed for automated structural classification of volatiles based on their IUPAC names (Djoumbou Feunang et al., 2016).

## Results and Discussion

### Multivariate Analysis of the Bacterial and Fungal Volatilomes

**FIGURE 1:**
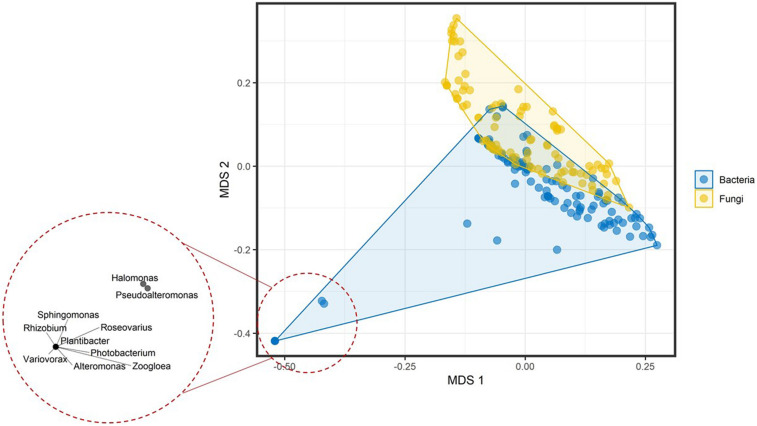
Multidimensional scaling plot of the volatilome profiles from bacterial and fungal genera differentiated at the domain (bacteria) and kingdom (fungi) level. The bottom left corner shows the clustering of the genera with very small volatilome sizes.

**FIGURE 2:**
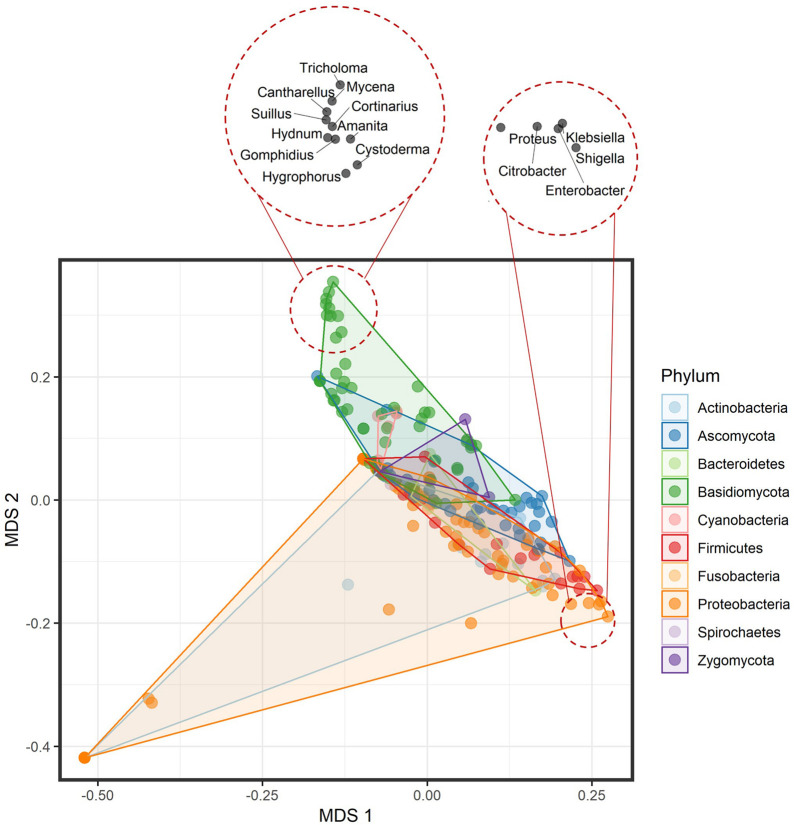
Multidimensional scaling plot of the volatilome profiles from bacterial and fungal genera differentiated at the phylum level. The top left corner shows the clustering of the Basidiomycota genera, while the bottom right shows the clustering of a few Proteobacteria genera.

Prepared mVOC dataset included 474 volatiles and 221 genera across the microbial tree of life and comprised 120 bacterial genera and 101 fungal genera. Multivariate data analysis of bacterial and fungal volatilomes showed differences at the kingdom (fungal) and domain (bacteria) level with *R*^2^ = 0.03751 and *P* = 0.001 by permutational multivariate analysis of variance (PERMANOVA) (Figure 1). At lower taxa levels, the explained variation increased and was highest at the family level with *R*^2^ = 0.72008 and *P* = 0.001 by PERMANOVA. This suggests that the optimum amount of variation in the volatilome profiles was explained more at the family level, which was 72% of the total variance. Volatilomes of *Alteromonas*, *Halomonas*, *Photobacterium*, *Plantibacter*, *Pseudoalteromonas*, *Rhizobium*, *Roseovarius*, *Sphingomonas*, *Variovorax*, and *Zoogloea* clustered separately at the bottom left corner of the multidimensional scaling (MDS) plot than the volatilomes of the other bacteria. After further analysis, it was revealed that these ten bacterial species were separated due to the very small volatilome size possibly due to the lack of investigations rather than the actual volatilome size or profile. Therefore, these bacteria should be investigated in more detail in the future to clarify whether the limited complexity of their volatilomes is a characteristic determinate. It can be speculated that these bacteria perform specialized metabolism due to their specialized ecological roles, e.g., *Rhizobium* species are symbiotic bacteria that act cooperatively with plant roots of the legume family; *Halomonas* species have been found in a variety of saline environments, including estuaries, ocean and saline lakes as it grows in the range of 5–25% NaCl; while *Zoogloea* cells form tree-like colonies/populations within a colloidal matrix indicating that their metabolism depends on individual cells and intercellular signal communication. At the phylum level, differences were also observed among microbial volatilome profiles with *R*^2^ = 0.12071 and *P* = 0.001 by PERMANOVA (Figure 2). This suggests that ∼12% of the data variance is explained by taxa classification at the phylum level. Several Proteobacteria genera (dark orange) (*Citrobacter*, *Enterobacter*, *Klebsiella*, *Proteus*, and *Shigella*) and Basidiomycota genera (dark green) (*Amanita*, *Cantharellus*, *Cortinarius*, *Cystoderma*, *Gomphidius*, *Hydnum*, *Hygrophorus*, *Mycena*, *Suillus*, and *Tricholoma*) were nicely separated from the bulk.

### Volatiles Frequency and Distribution Among Bacterial and Fungal Genera

Among all volatilomes, 34 volatiles were produced by at least 10% of all included genera (Table 1). Numerous volatiles such as dimethyl disulfide, acetic acid, 2-nonanone, dimethyl trisulfide, 2-undecanone, isovaleric acid, 2-tridecanone, propanoic acid, and indole were produced by a significant number of bacterial genera (>75%) rather than by fungal genera. These compounds can be characterized as “typical bacterial VOCs,” and literature shows that they have been documented to be produced by bacterial species (Bos et al., 2013; Fincheira and Quiroz, 2018; Caspani et al., 2019).

**TABLE 1:** Volatiles that are produced by at least 10% of the bacterial and fungal genera.

Volatile	Chemical class	Number of genera	Genera %	Bacterial genera %	Fungal genera %
3-Methyl-1-butanol	Alcohol	71	32.1	54.9	45.1
2-Phenylethanol	Aromatic compound, alcohol	57	25.8	63.2	36.8
Dimethyl disulfide	Organosulfur compound	52	23.5	**86.5**	13.5
Benzaldehyde	Aromatic compound, aldehyde	51	23.1	66.7	33.3
Acetic acid	Carboxylic acid	40	18.1	**82.5**	17.5
2-Methyl-1-butanol	Alcohol	39	17.6	35.9	64.1
2-Nonanone	Ketone	39	17.6	76.9	23.1
1-octen-3-ol	Alcohol	37	16.7	8.1	**91.9**
Dimethyl trisulfide	Organosulfur compound	37	16.7	**89.2**	10.8
Isobutanol	Alcohol	37	16.7	37.8	62.2
Pinene	Monoterpene	37	16.7	37.8	62.2
2-undecanone	Ketone	35	15.8	77.1	22.9
Ethanol	Alcohol	34	15.4	47.1	52.9
Isovaleric acid	Methyl-branched fatty acid	34	15.4	**79.4**	20.6
Limonene	Monoterpene	32	14.5	40.6	59.4
Butanone	Ketone	31	14.0	71.0	29.0
2-heptanone	Ketone	30	13.6	60.0	40.0
3-octanone	Ketone	30	13.6	13.3	**86.7**
Acetophenone	Ketone	29	13.1	65.5	34.5
Acetoin	Ketone	28	12.7	67.9	32.1
Benzyl alcohol	Aromatic compound, Alcohol	27	12.2	74.1	25.9
2-pentanone	Ketone	26	11.8	61.5	38.5
2-tridecanone	Ketone	25	11.3	96.0	4.0
Decanal	Aldehyde	25	11.3	64.0	36.0
1-hexanol	Alcohol	24	10.9	41.7	58.3
1-octanol	Alcohol	24	10.9	50.0	50.0
Dimethyl sulfide	Organosulfur compound	24	10.9	**75.0**	25.0
Propanoic acid	Carboxylic acid	24	10.9	**91.7**	8.3
Nonanal	Aldehyde	23	10.4	60.9	39.1
1-butanol	Alcohol	22	10.0	54.5	45.5
2-pentylfuran	Heterocyclic compound	22	10.0	9.1	**90.9**
Acetone	Ketone	22	10.0	59.1	40.9
Indole	Heterocyclic compound	22	10.0	77.3	22.7
Phenylacetaldehyde	Aromatic compound, aldehyde	22	10.0	59.1	40.9

As a case study, we considered dimethyl disulfide and dimethyl trisulfide, which are byproducts derived from methionine amino acid degradation. This process is catalyzed by the enzyme L-methionine γ-lyase that carries the conserved domain PRK06234 (Hanniffy et al., 2009; Marchler-Bauer et al., 2017). All proteins registered in the National Center for Biotechnology Information (NCBI) Reference Sequence (RefSeq) database that harbor the same domain structure (Marchler-Bauer et al., 2017) were identified. Among 552,557 proteins, 94.44% of the protein sequences belonged to the species in the bacterial domain, while 5.56% belonged to the fungal kingdom – after normalization to the total number of bacterial and fungal protein sequences in RefSeq Release 93. Genomic data supports volatilome analysis regarding the prevalence of sulfur amino acid metabolism in the majority of bacterial species to generate respective VOCs. It appears that the volatile sulfur metabolism is dominated by bacteria rather than by fungi, which indicates a differential impact on the ecological roles of both phyla. The volatilomes, however, may be biased, as growth media used in *in vitro* culturing influence microbial metabolism and respective product profiles, including the volatilomes. In the lab, bacteria are usually grown on Luria-Bertani (LB) agar or nutrient broth (NB) agar complex media which are protein-rich, while the typical fungal medium is potato dextrose agar (PDA), which is carbon-rich (Velez et al., 2018).

Moreover, a fewer number of volatiles were produced mainly (>75%) by fungal rather than bacterial genera such as 1-octen-3-ol, 3-octanone, and 2-pentylfuran and can be considered common fungal VOCs (Table 1). Interestingly, 1-octen-3-ol, 3-octanone, and 2-pentylfuran are known as lipid peroxidation and degradation products of linoleic acid in *Aspergillus* species, e.g., *A. fumigatus* and *A. flavus* (Heddergott et al., 2014; Miyamoto et al., 2014). Whether 1-octen-3-ol, 3-octanone, and 2-pentylfuran serve any function to these fungi has yet to be determined. 2-Pentylfuran was proposed as a marker for *A. fumigatus* in the breath of lung-infected patients, however, this was not further pursued due to non-specificity (Chambers et al., 2009; Mercier et al., 2018). One of the gene families involved in this reaction is the fatty acid dioxygenase, which harbors two conserved domains: cd09817 [linoleate (8R)-dioxygenase and related enzyme] and COG2124 (cytochrome P450). A genomic analysis with these conserved domains showed that among 393 proteins, 99.84% belonged to the fungal kingdom, while 0.16% belonged to the bacterial domain after normalization to the total number of fungal and bacterial proteins in RefSeq Release 93 (O’Leary et al., 2016). This mirrors the volatilome data as the majority of the genera producing those fatty acid-derived volatiles were from strains of the fungal kingdom.

**FIGURE 3:**
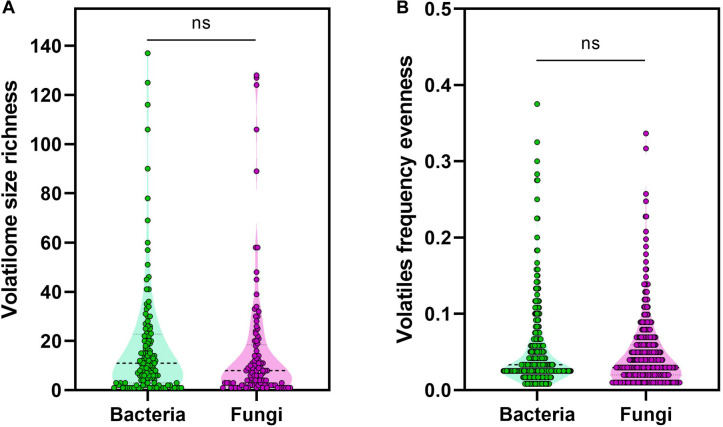
Volatiles richness and evenness. **(A)** Volatilome size richness was calculated as the total number of volatiles produced by a genus, **(B)** while volatiles frequency was calculated as the division of the number of genera producing a volatile by the total number of genera investigated. The non-parametric Mann–Whitney *U*-test was used to test for statistical significance.

To ensure that the calculated distribution patterns are not due to skewness in the datasets size, datasets were compared in terms of the volatilome richness or evenness. This richness and evenness comparison revealed no statistically significant difference between the volatilome size of the bacterial and fungal genera (Figure 3A). Moreover, no statistically significant difference between the evenness of the produced volatiles by bacterial and fungal genera was detected (Figure 3B). It is noticeable that the median of volatilome size was ∼10 volatiles, however, some genera exhibited a relatively huge volatilome size reaching up to 140 volatiles. This is again attributed to the extensive investigation of the VOC profiles of several bacteria and fungi that are important for medical, agricultural, or economical reasons. The genera are ranked in a descending order according to their volatilome size: *Pseudomonas*, *Tuber*, *Penicillium*, *Serratia*, *Aspergillus*, *Bacillus*, *Streptomyces*, *Fusarium*, *Escherichia*, *Trichoderma*, and *Staphylococcus*. Overall, our analyses did not show any difference in the diversity of the bacterial and fungal volatilomes.

### Volatiles With a Strong Phylogenetic Signal

**FIGURE 4:**
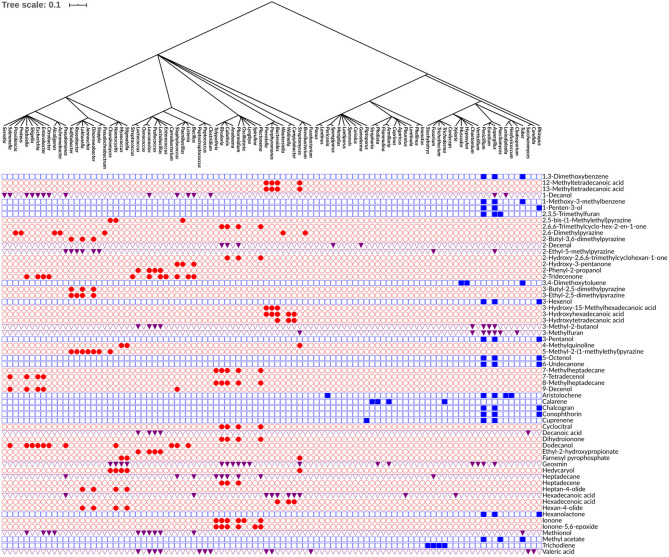
Phylogenetic tree of bacterial and fungal genera annotated with the 61 phylogenetically conserved volatiles. Rows indicate the distribution of each volatile across the bacterial and fungal genera. Red circles represent the volatiles that are phylogenetically conserved among bacterial genera only. Blue squares represent the volatiles that are phylogenetically conserved among fungal genera only. Purple triangles represent the volatiles that are phylogenetically conserved among both bacterial and fungal genera. The production of volatiles is visualized in a binary fashion, filled or empty symbols indicate that a volatile is present or absent, respectively. The phylogenetic tree was visualized and annotated using the Interactive Tree of Life (iTOL) version 5.6.1.

Among the 474 volatiles, 61 exhibited a significant phylogenetic signal as demonstrated by its *P*-value (Tables 2–4 and Figure 4). For example, for a volatile to have a significant phylogenetic signal, its corresponding phylogenetic *D* statistic *P*-value should be <0.05 (Fritz and Purvis, 2010). This means that a volatile is phylogenetically conserved more than expected under Brownian threshold model. 33 volatiles were phylogenetically conserved in the bacterial domain (Table 2 and Figure 4), whereas 17 volatiles were phylogenetically conserved in the fungal kingdom (Table 3 and Figure 4), and 11 volatiles were phylogenetically conserved in genera from both bacterial domain and fungal kingdom (Table 4 and Figure 4).

**TABLE 2:** Phylogenetically conserved volatiles in genera belonging to bacterial domain only.

Volatile	Chemical class	*D* Estimate	Phylogenetic signal	*P*-value phylogenetic	*P*-value Brownian	Number of bacterial genera
3-Hydroxy-15-methylhexadecanoic acid	Long-chain fatty acid	−4.12578	5.125784	0	0.996	3
Hedycaryol	Sesquiterpene	−2.35126	3.351256	0	0.985	5
3-hydroxyhexadecanoic acid	Long-chain fatty acid	−2.24339	3.243395	0	0.988	5
12-methyltetradecanoic acid	Long-chain fatty acid	−2.04942	3.049425	0	0.971	4
13-methyltetradecanoic acid	Long-chain fatty acid	−2.01785	3.017848	0	0.975	4
7-tetradecenol	Alcohol	−1.90478	2.904777	0	0.943	4
Heptadecene	Alkene	−1.8946	2.894599	0	0.943	3
2,6,6-trimethylcyclo-hex-2-en-1-one	Ketone	−1.78744	2.78744	0	0.948	4
Ionone	Heterocyclic compound	−1.77631	2.776307	0	0.981	6
Dihydroionone	Heterocyclic compound	−1.73277	2.732767	0	0.943	4
Cyclocitral	Monoterpene	−1.69961	2.699614	0	0.956	4
3-hydroxytetradecanoic acid	Long-chain fatty acid	−1.68834	2.688339	0	0.895	3
Ionone-5,6-epoxide	Heterocyclic compound	−1.68477	2.684775	0	0.975	6
Ethyl-2-hydroxypropionate	Ester	−1.68138	2.681376	0	0.942	4
2-phenyl-2-propanol	Aromatic compound, alcohol	−1.68063	2.680632	0	0.938	4
8-methylheptadecane	Alkane	−1.60584	2.605841	0	0.963	5
Hexadecenoic acid	Long-chain fatty acid	−1.53466	2.534657	0.001	0.887	3
7-methylheptadecane	Alkane	−1.53265	2.532646	0	0.951	5
5-methyl-2-(1-methylethyl)pyrazine	Heterocyclic compound	−1.02303	2.023027	0	0.921	7
2-hydroxy-3-pentanone	Ketone	−1.02267	2.022671	0.002	0.792	3
9-decenol	Unsaturated alcohol	−0.94583	1.945835	0	0.862	5
3-ethyl-2,5-dimethylpyrazine	Heterocyclic compounds	−0.93737	1.937368	0	0.823	4
Dodecanol	Alcohol	−0.74702	1.747024	0	0.912	11
2-butyl-3,6-dimethylpyrazine	Heterocyclic compound	−0.62766	1.627655	0.005	0.753	3
2-hydroxy-2,6,6-trimethylcyclohexan-1-one	Ketone	−0.58202	1.58202	0.007	0.746	3
3-butyl-2,5-dimethylpyrazine	Heterocyclic compound	−0.56563	1.565633	0.01	0.737	3
2-tridecenone	Ketone	−0.37951	1.379511	0	0.789	11
4-methylquinoline	Heterocyclic compound	−0.30888	1.308876	0.014	0.701	3
Farnesyl pyrophosphate	Prenyl pyrophosphate	−0.22603	1.226032	0.011	0.689	3
2,5-bis-(1-methylethyl)pyrazine	Heterocyclic compound	−0.19579	1.195791	0.015	0.653	3
2,6-dimethylpyrazine	Heterocyclic compound	−0.17457	1.174567	0	0.649	7
Heptan-4-olide	Lactone	−0.04932	1.04932	0.008	0.617	4
Hexan-4-olide	Lactone	−0.02547	1.025475	0.009	0.585	4

**TABLE 3:** Phylogenetically conserved volatiles in genera belonging to fungal kingdom only.

Volatile	Chemical class	*D* Estimate	Phylogenetic signal	*P*-value phylogenetic	*P*-value brownian	Number of fungal genera
Trichodiene	Unsaturated hydrocarbon	−1.95004	2.950041	0	0.961	4
2,3,5-trimethylfuran	Heterocyclic compound	−1.59661	2.596614	0.005	0.851	3
3,4-dimethoxytoluene	Aromatic compound	−0.74437	1.74437	0.007	0.776	3
Aristolochene	Sesquiterpene	−0.73614	1.736145	0	0.835	5
1,3-dimethoxybenzene	Aromatic compound	−0.54314	1.543142	0.005	0.731	3
Calarene	Sesquiterpene	−0.53914	1.539141	0.001	0.748	4
1-methoxy-3-methylbenzene	Aromatic compound	−0.49196	1.491962	0.01	0.728	3
3-hexenol	Alcohol	−0.35565	1.355653	0.011	0.718	3
6-undecanone	Ketone	−0.3359	1.335898	0.007	0.709	3
5-octenol	Alcohol	−0.31986	1.319864	0.014	0.697	3
3-pentanol	Alcohol	−0.29544	1.295439	0.003	0.698	3
1-penten-3-ol	Alcohol	−0.2864	1.286402	0.004	0.703	3
Hexanolactone	Lactone	−0.26708	1.267075	0.007	0.688	3
Chalcogran	Ketal	−0.26474	1.264744	0.007	0.691	3
Conophthorin	Ketal	−0.26047	1.260467	0.007	0.694	3
Cuprenene	Sesquiterpene	−0.18634	1.186336	0.008	0.649	3
Methyl acetate	Ester	−0.03907	1.039073	0.021	0.602	3

**TABLE 4:** Phylogenetically conserved volatiles in genera belonging to both bacterial domain and fungal kingdom.

Volatile	Chemical class	*D* Estimate	Phylogenetic signal	*P*-value phylogenetic	*P*-value Brownian	Number of bacterial genera	Number of fungal genera
Decanoic acid	Medium-chain fatty acid	−0.78737	1.787369	0	0.832	4	1
3-methyl-2-butanol	Alcohol	−0.5385	1.538499	0	0.828	4	4
3-methylfuran	Heterocyclic compound	−0.42748	1.427482	0	0.759	1	6
2-decenal	Aldehyde	−0.26139	1.261393	0	0.674	3	2
Valeric acid	Straight chain fatty acid	−0.26112	1.261118	0	0.721	10	2
1-decanol	Alcohol	−0.24541	1.245407	0	0.707	13	2
Methionol	Organosulfur compound	−0.17737	1.177371	0	0.671	10	1
Geosmin	Alcohol	−0.1643	1.164295	0	0.684	11	6
Heptadecane	Saturated hydrocarbon	−0.10067	1.100673	0	0.613	8	1
Hexadecanoic acid	Long-chain fatty acid	−0.04772	1.047717	0.001	0.586	8	2
2-ethyl-5-methylpyrazine	Heterocyclic compound	−0.01893	1.018933	0.003	0.564	6	2

One of the identified volatiles with a strong phylogenetic signal in the bacterial genera was the monoterpene cyclocitral. The present analysis indicated that its production is phylogenetically conserved within the cyanobacteria phylum by the genera: *Calothrix*, *Rivularia*, *Plectonema*, and *Phormidium*. This result matches previous knowledge that cyclocitral is exclusively produced by cyanobacteria (Harada et al., 2009). It causes a color change from green to blue during cell lysis (Harada et al., 2009). Cyclocitral exhibits a strong inhibitory activity against cyanobacteria (Arii et al., 2015) in what is known as a grazer defense signal, which is unique to the cyanobacterium *Microcystis* (Jüttner et al., 2010). It acts as chemical signal of an unsuitable food organism and a repellent to predator/grazer, therefore protecting *Microcystis* colonies (Jüttner et al., 2010). Monoterpene biosynthesis and emission is a prerequisite in many plant species, while so far only a few monoterpene synthases and ca. 55 sesquiterpene synthases have been isolated from bacteria (Dickschat, 2016). Therefore, it is not surprising that hedycaryol, ionone and dihydroionone were identified as strong phylogenetic signals in the present analysis. Hedycaryol acts as a defense compound in plants (Liang et al., 2018), while biological and ecological roles of the bacterial hedycaryol remains unknown. Ionone and dihydroionone are aromatic compounds with a great interest to fragrance industry, however, their functional role have yet not been described in bacteria (Zhang et al., 2018). Furthermore, it is interesting to note that in bacteria several long fatty acids, pyrazines, and their derivatives are strong phylogenetic signals. Pyrazines are typically biosynthesized by bacteria, e.g., 3,5-dimethyl pyrazine was recently proposed to be a new quorum sensing signal playing an important role in commensal and pathogenic bacteria, such as *Vibrio cholerae* (Papenfort et al., 2017).

Aristolochene, a bicyclic volatile sesquiterpene, was found as another phylogenetically conserved volatile (Table 3). Aristolochene is produced by the fungal genera *Aspergillus*, *Penicillium*, *Neofusicoccum*, *Lasiodiplodia*, and *Periconia*. It is biosynthesized from farnesyl pyrophosphate by aristolochene synthase (Proctor and Hohn, 1993). Aristolochene is a sesquiterpene that is a precursor for mycotoxins such as the PR toxin produced by *Penicillium roqueforti* (Jeleń, 2002; Dubey et al., 2018). Aristolochene was detected alongside the PR toxin and therefore it was proposed as a marker for the PR toxin (Jeleń, 2002; Dubey et al., 2018). The biosynthetic cluster for the production of PR toxin and its precursors was recently characterized (Hidalgo et al., 2014, 2017). Moreover, it was shown that disruption in that biosynthetic cluster led to overproduction of mycophenolic acid, an antitumor compound confirming the role of aristolochene and other PR toxin precursors as cell signaling molecules (Hidalgo et al., 2014, 2017). In order to confirm the presence of the biosynthetic pathway of aristolochene in one of the genera we identified and visualize its biosynthetic pathway, we used iPath 3.0: interactive pathways explorer v3 tool (Darzi et al., 2018). We chose *Neofusicoccum parvum* as an example for an aristolochene-producing species and *Escherichia coli* as a non-producer. Then, we mapped the KEGG Orthology entries (KOs) of both species to the biosynthesis of secondary metabolites pathways. We showed that only *N. parvum* has the complete pathway for the biosynthesis of aristolochene, in comparison to *E. coli* (Supplementary Figure 1A).

Geosmin is a typical compound biosynthesized by bacteria as well as fungi (Table 4), which include various bacterial genera (i.e., *Streptomyces*, *Anabaena*, *Calothrix*, *Rivularia*, *Lyngbya*, *Oscillatoria*, *Phormidium*, *Stigmatella*, *Myxococcus*, *Nannocystis*, and *Chondromyces*) and some fungal genera (i.e., *Aspergillus*, *Penicillium*, *Verticillium*, *Chaetomium*, *Armillaria*, and *Pholiota*). Its earthy smell is characteristic and well known and can easily be recognized by humans in different environments (e.g., humid forests). Although this volatile is known for quite a long time, the biological or ecological role remains mostly elusive. It was suggested that it functions as an indicator of contaminated food as well as water (Stensmyr et al., 2012). Similar to aristolochene, we wanted to confirm the presence of the biosynthetic pathway of geosmin in one of the genera we identified and visualize its biosynthetic pathway using iPath 3.0 (Darzi et al., 2018). We chose *Streptomyces albidoflavus* as an example for a propanoic acid-producing species and *E. coli* as a non-producer. Then, we mapped the KEGG Orthology entries (KOs) of both species to the metabolic pathways. We showed that only *S. albidoflavus* has the complete pathway for the biosynthesis of propanoic acid, in comparison to *E. coli* (Supplementary Figure 1B).

3-Hydroxy-15-methylhexadecanoic acid was among the fatty acids identified in this study with a strong phylogenetic signal. It was only observed in bacterial genera that belong to the same phylum, Bacteroidetes (e.g., *Bacteroides*, *Porphyromonas*, and *Prevotella*). 3-Hydroxy-15-methylhexadecanoic acid is one of the characteristic lipid A components of the lipopolysaccharides (LPS) (Johne and Bryn, 1986; Ogawa, 1993). Whether this fatty acid is critical for the endotoxicity of these Gram-negative bacteria is not well-defined.

In summary, our phylogenetic signal testing revealed that the production of several volatiles is phylogenetically conserved. This work gives a new framework for studying microbial volatiles by integrating metabolomic and phylogenetic data. The presented approach of phylogenetic signal testing does not give a clue whether the genes for the biosynthesis of volatiles are vertically or horizontally transferred between species during evolution.

### Human Volatiles Versus Microbial Volatiles

**FIGURE 5:**
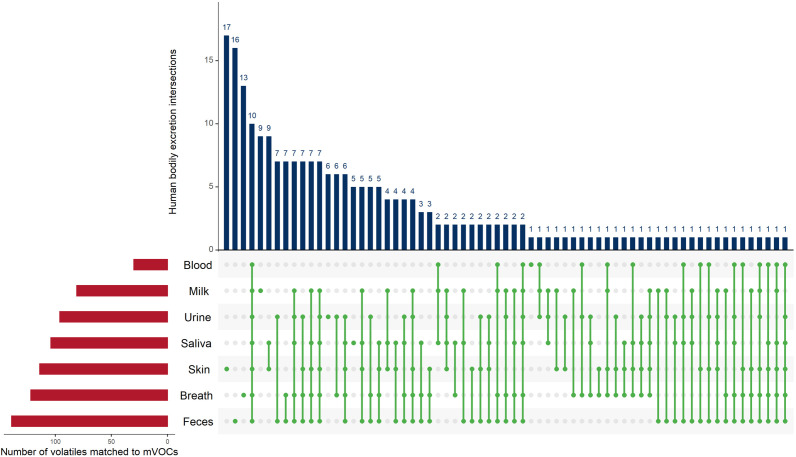
UpSet plot of the intersections of the 229 volatiles identified from human bodily excretions that shared with microbial volatiles. The red bars **(bottom left)** indicate the number of microbial volatiles matching human volatiles from each bodily excretion. The green dots (connected with green lines) represent the intersections of the volatiles in bodily excretions and the blue bars **(top)** indicate the frequency of these intersections. For example, the fourth blue bar from the left shows 10, which is the number of volatiles detected from all bodily excretions (which is indicated by the green dotes across all bodily excretions). The UpSet plot was generated using the *UpSetR* R package, https://cran.r-project.org/web/packages/UpSetR/ (Conway et al., 2017).

de Lacy Costello et al. (2014) estimated that the human body emits 1,840 volatiles. These volatiles were detected from different bodily fluids or excretions (i.e., feces, urine, breath, skin secretions, milk, blood, and saliva). The human microbiome is, with no surprise, an important contributor to the human metabolic capacity and the production of several of metabolites and volatiles (Visconti et al., 2019; Elmassry and Piechulla, 2020). However, the number of shared human (hVOCs) and microbial volatiles is not known. We hypothesized that many shared hVOCS and mVOCs exist. Therefore, we compared the 476 mVOCs analyzed in this study (this does not represent all microbial volatiles) and the hVOCs. Indeed, we found that out of the 476 mVOCs 229 volatiles (∼48%) were also produced in/from humans (Figure 5). Human fecal volatilome ranked the first in terms of the number of overlapping volatiles with mVOCs, ∼139 volatiles (Figure 5). This is not surprising because the human gut harbors the largest number and most diverse microbiota in the human body (Almeida et al., 2019; Integrative Hmp (iHMP) Research Network Consortium, 2019). It is presumed that many of those hVOCS detected from feces originate from the gut microbiota itself due to their massive metabolic capacity. Moreover, blood hVOCS showed the least resemblance to mVOCs (Figure 5). Finally, we found 10 hVOCs common to all bodily excretions and mVOCs, i.e., 1-butanol, acetaldehyde, acetone, benzaldehyde, heptanal, hexanal, octanal, pentanol, styrene, and toluene (Figure 5). However, further investigation is required to determine whether the shared hVOCs and mVOCs are produced exclusively by our human microbiome.

## Conclusion

Microbial VOCs are integral components of the microbial metabolome. They serve numerous biological and ecological functions in plants, microbes, and animals (Piechulla et al., 2017; Piechulla et al., 2020), covering a wide range of action potentials from signaling to virulence. However, many mVOCs have unknown functions yet to be discovered. Across 221 bacterial and fungal genera, it was observed that the production of 61 mVOCs was phylogenetically conserved. Many of which were dominantly emitted either of bacterial domain or fungal kingdom and are considered common bacterial VOCs (e.g., dimethyl disulfide and acetic acid) or common fungal VOCs (e.g., 1-octen-3-ol and 2-pentylfuran), respectively and maybe used as fingerprints or biomarkers. This analysis shed light on several underestimated volatiles that could have potential in various applications. These applications concern basic sciences (i.e., to understand the biological or ecological roles of these volatiles in microorganismal communities) as well as applied research (i.e., to utilize these volatiles in diagnostics of pathogenic microorganisms or to utilize the respective microorganisms to produce specific volatiles in industry using biotechnology). We note that our results should be considered carefully as they were based on a rather qualitative than quantitative dataset. Also, our analysis was limited to available data on only 221 genera. Therefore, the true diversity in the metabolic capacity of the microbial life may not be represented by our investigation.

## Data Availability Statement

The data analyzed in this study is subject to the following licenses/restrictions: the dataset used in this study are compiled in mVOC 2.0 database (http://bioinformatics.charite.de/mvoc/). The raw data for the bioinformatics analysis used are available upon request. Requests to access these datasets should be directed to ML, marie.lemfack@uni-rostock.de; BP, birgit.piechulla@uni-rostock.de.

## Author Contributions

BP and ML provided the data. ME performed data analysis and drafting the work. ME and MF originally produced preliminary hypotheses. ME, MF, BP, and ML critically revised and interpreted the results of the study. RP, B-OG, BP, and ML helped revise the manuscript critically for important intellectual content. All authors reviewed, revised, and approved the final version of the manuscript.

## Conflict of Interest

The authors declare that the research was conducted in the absence of any commercial or financial relationships that could be construed as a potential conflict of interest.

## Footnotes

1 http://bioinformatics.charite.de/mvoc

2 http://bioinformatics.charite.de/mvoc

3 https://r-project.org

4 https://arb-silva.de

5 https://cran.r-project.org/web/packages/phylobase

6 https://cran.r-project.org/web/packages/vegan

7 https://cran.r-project.org/web/packages/magrittr

8 https://cran.r-project.org/web/packages/dplyr

9 https://cran.r-project.org/web/packages/ggpubr

10 https://cran.r-project.org/web/packages/caper

11 https://pubchem.ncbi.nlm.nih.gov

12 http://classyfire.wishartlab.com

## Supplementary Material

Supplementary Figure 1 Overview of the biosynthetic pathways for aristolochene and geosmin. **(A)** Comparative biosynthesis of secondary metabolites pathways based on KEGG Orthology entries (KOs) between *Neofusicoccum parvum* [aristolochene (yellow circle) producer] and *Escherichia coli.* **(B)** Comparative biosynthesis of secondary metabolites pathways based on KOs between *Streptomyces albidoflavus* [propanoic acid (yellow circle) producer] and *E. coli.* Maps were visualized by iPath3.0: interactive pathways explorer v3.
