# Supplementary figures and images for: Sixty-One Volatiles Have Phylogenetic Signals Across Bacterial Domain and Fungal Kingdom
Source: Front Microbiol. 2020 Sep 30;11:557253. doi: 10.3389/fmicb.2020.557253 (PMC7554305; doi:10.3389/fmicb.2020.557253)

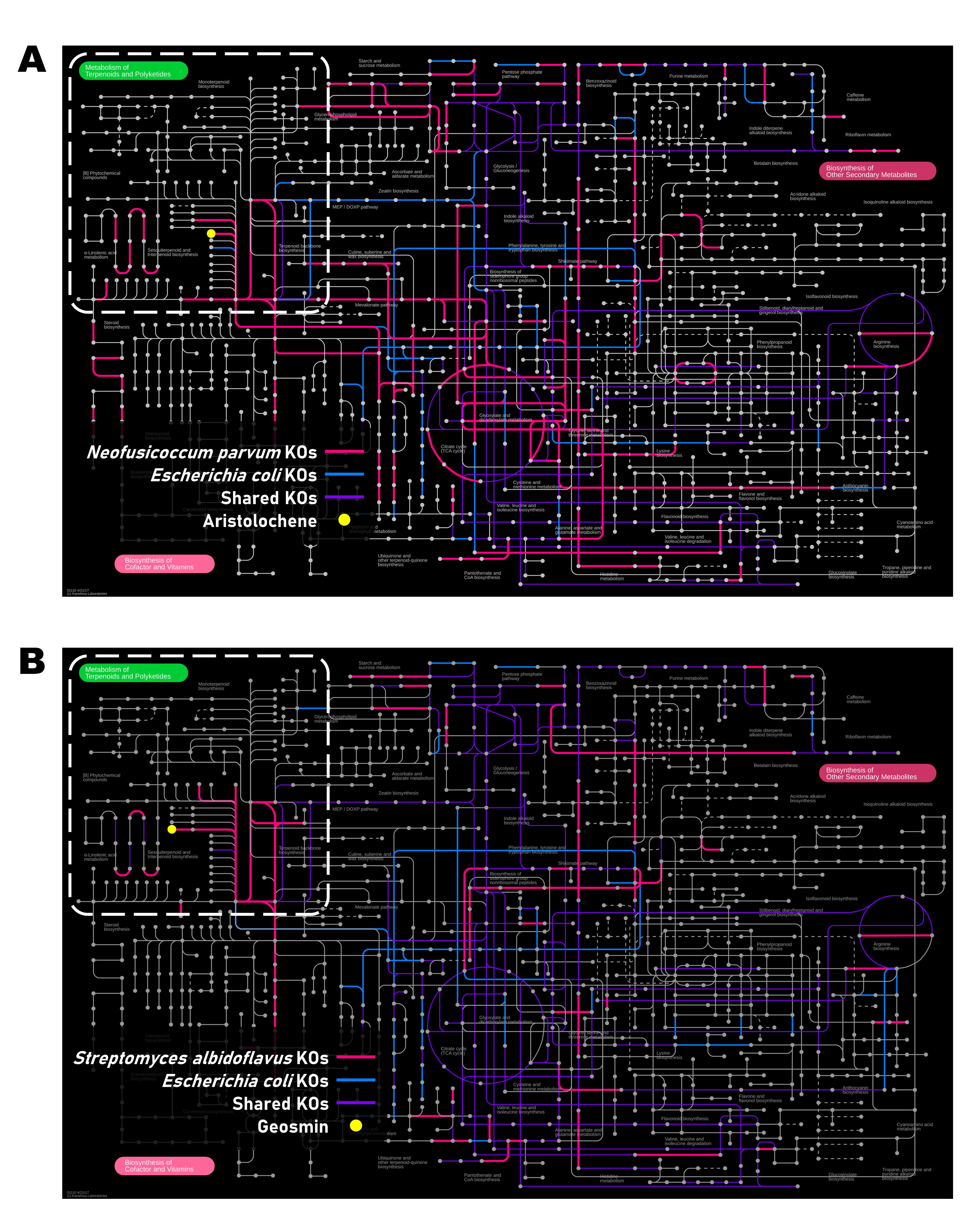

Supplement: Supplementary Figure 1 — Overview of the biosynthetic pathways for aristolochene and geosmin. (A) Comparative biosynthesis of secondary metabolites pathways based on KEGG Orthology entries (KOs) between Neofusicoccum parvum [aristolochene (yellow circle) producer] and Escherichia coli. (B) Comparative biosynthesis of secondary metabolites pathways based on KOs between Streptomyces albidoflavus [propanoic acid (yellow circle) producer] and E. coli. Maps were visualized by iPath3.0: interactive pathways explorer v3. [file Image_1.JPEG]
